# Immune signatures for HIV-1 and HIV-2 induced CD4^+^T cell dysregulation in an Indian cohort

**DOI:** 10.1186/s12879-019-3743-7

**Published:** 2019-02-11

**Authors:** Sukeshani Salwe, Amitkumar Singh, Varsha Padwal, Shilpa Velhal, Vidya Nagar, Priya Patil, Alaka Deshpande, Vainav Patel

**Affiliations:** 1 Department of Biochemistry and Virology, National Institute for Research in Reproductive Health, Indian Council of Medical Research, J. M. Street, Parel, Mumbai, 400012 India; 2 Department of Medicine, Grant Medical College & Sir J. J. group of Hospitals, Mumbai, 400008 India

**Keywords:** HIV-2, Anti-retroviral therapy, Regulatory T cells, CD4^+^T-cell subsets, Immune activation, Cytotoxic T cells, Granzyme B, Immune dysregulation, Longitudinal analysis

## Abstract

**Background:**

HIV-2 infection is characterised by a longer asymptomatic phase and slower AIDS progression than HIV-1 infection. Identifying unique immune signatures associated with HIV-2 pathogenesis may thus provide therapeutically useful insight into the management of HIV infection. This study examined the dynamics of the CD4^+^T cell compartment, critical in disease progression, focussing on chronic HIV-2 and HIV-1 infected individuals at various stages of disease progression.

**Methods:**

A total of 111 participants including untreated and treated HIV infected individuals and seronegative individuals were enrolled in this study. The relative proportion of CD4^+^T cell subsets, expressing CD25 (IL-2Rα) and CD127 (IL-7R), in HIV infected individuals and seronegative controls were assessed by multiparametric flow cytometry. Additionally, levels of immune activation and cytotoxic T lymphocytes in both the CD4^+^T and CD8^+^T cell compartments was evaluated.

**Results:**

Both treated and untreated, HIV-1 and HIV-2 infected individuals showed apparent dysregulation in CD4^+^ T cell subset frequency that was associated with disease progression. Furthermore, longitudinal sampling from a group of HIV-1 infected individuals on virologically effective ART showed no significant change in dysregulated CD4^+^T cell subset frequency. For both ART naïve and receiving groups associations with disease progression were strongest and significant with CD4^+^ T cell subset frequency compared to per cell expression of IL-2Rα and IL-7Rα. In untreated HIV-2 infected individuals, T cell activation was lower compared to ART naïve HIV-1 infected individuals and higher than seronegative individuals. Also, the level of Granzyme-B expressing circulating T cells was higher in both ART-naïve HIV-1 and HIV-2 infected individuals compared to seronegative controls.

**Conclusion:**

Dysregulation of IL-2 and IL-7 homeostasis persists in CD4^+^T cell subsets irrespective of presence or absence of viremia or antiretroviral therapy in HIV infection. Furthermore, we report for the first time on levels of circulating Granzyme-B expressing CD4^+^T and CD8^+^T cells in chronic HIV-2 infection. Lower immune activation in these individuals indicates that persistent immune activation driven CD4^+^T cell depletion, as observed in untreated HIV-1 infected individuals, may not be as severe and provides evidence for a disparate pathogenesis mechanism. Our work also supports novel immunomodulatory therapeutic strategies for both HIV-1 and HIV-2 infection.

**Electronic supplementary material:**

The online version of this article (10.1186/s12879-019-3743-7) contains supplementary material, which is available to authorized users.

## Background

An estimated total of 36.7 million [34.0 million–39.8 million] individuals worldwide, are people living with human immunodeficiency virus (PLHIV) [1].Of these, around 2.1 million [1.71 million–2.64 million] are estimated to live in India [2].The HIV epidemic in India largely involves sexual transmission of HIV-1 Clade C viruses [3]. Furthermore, HIV-2 single infection and dual (HIV-1 along with HIV-2) infection is clearly present and persistent in the HIV infected population in India [4, 5]. Both HIV-1 and HIV-2 share the same modes of transmission, namely sexual contact, blood-borne exposure (blood transfusion, shared needles) and perinatal transmission. Also, both infections are associated with a progressive decline of CD4^+^T cells and loss of immune function, which manifests clinically as an increased susceptibility to opportunistic infections. However, the pathogenesis associated with these two viruses is reported to be disparate. HIV-2 infection has been associated with a significantly longer asymptomatic stage, lower plasma viral load, slower decline in CD4^+^T-cell count, and lower mortality rate attributable to AIDS compared to HIV-1 [6–8]. In addition, a well-preserved and polyfunctional HIV-specific memory CD4^+^T cell response has been shown to be a hall mark of HIV-2 infection [9, 10].

Thus delineating unique immune signatures associated with HIV-2 pathogenesis may provide therapeutically useful insight into the management of HIV-1 infection and AIDS. The CD4^+^T cell compartment is critical in disease progression as it serves both, as a target and also as a marker of disease progression. Also, CD4^+^ T cell help is an important component of immune responses, both cellular and humoral. In this study, we examined the dynamics of the CD4^+^ T cell compartment in terms of various important subsets, based on homeostatic markers, focussing on chronic HIV-1 and HIV-2 infected individuals at various stages of disease progression.

To address homeostatic turnover, CD4^+^T cell subsets have been defined with respect to expression of Interleukin-2 (IL-2) and Interleukin-7 (IL-7) receptors [11]. The receptors for IL-2 and IL-7 share the common γ-chain in combination with unique high-affinity receptors: IL-2Rα (CD25) or IL-2Rβ (CD122) in the case of IL-2 and IL-7Rα (CD127) in that for IL-7 [12]. IL-2 and IL-7 function through their receptors as master regulators of T cell development, naïve and memory T cell homeostasis, proliferation, and differentiation [12–14]. CD4^+^ T cells can be categorized into three subsets on the basis of expression of IL2Rα (CD25) and IL7R (CD127): CD127^+^CD25^low/−^, CD127^−^CD25^−^, and CD25^high^CD127^low^ as previously reported [11]. This report characterized these phenotypes for the state of differentiation (i.e., naive vs. central memory vs. effector memory) using CD45RA and CD62L in HIV-1 and seronegative individuals. It was observed that CD127^+^CD25^low/−^ subset corresponded to both CD45RA^+^CD62L^+^ naive cells and CD45RA^−^ CD62L^+^ central memory cells. The CD127^−^ CD25^−^ subset consisted of mainly CD45RA^−^CD62L^−^ effector memory cells. The CD25^high^CD127^low^ phenotype of Regulatory T cells (Treg) has been defined by various study groups [15, 16] and was validated to correspond to CD4^+^CD25^+^Foxp3^+^ Treg cells also by us (Additional file 1: Figure S1). As, the dynamics of the CD4^+^T cell compartment may differ based on the presence or absence of active viral replication, we have evaluated the relative proportions of three subsets of CD4^+^ T cells that are defined based on expression of CD127 and CD25 under anti-retroviral therapy (ART) naïve and therapy receiving conditions in both HIV-1 and HIV-2 infected individuals. Furthermore, some HIV-1 infected individuals were followed longitudinally to assess the effect of long term ART on the dynamics of CD4^+^T cell compartment.

Systemic chronic immune activation is considered as the driving force of CD4^+^T cell depletion and resulting disease progression following HIV infection [17, 18], making it an important cellular parameter to evaluate in the context of HIV-1 and HIV-2 infection. Also, cytotoxic T cells (CTL) expressing the effector molecule Granzyme B (GrzB) are central in conferring host cell immunity against viral pathogens through recognition and killing of infected cells [19–22]. Thus, this study was designed, to examine levels of activation as well as Granzyme B expression in the both CD4^+^T and CD8^+^ T cells in chronic HIV infection and to delineate any unique immune signatures specific to either HIV-1 or HIV-2 infection.

## Methods

### Study subjects

A total 111 individuals were recruited for this cross sectional study including 33 seronegative, 24 antiretroviral therapy (ART) naïve HIV-1infected individuals, 16 ART-naïve HIV-2 infected individuals,19 ART-receiving HIV-1 and 19 ART- receiving HIV-2. All the participants were recruited from ART Centres, in Maharashtra with approval from the NIRRH Institutional Clinical Ethics Committee (Project no.:225/2012) and informed consent was obtained from all of them. The clinical features of the enrolled participants (age, viral load, absolute CD4 count and status of antiretroviral therapy) are shown in Table 1. Total nucleic acid from blood was isolated using the MagNa pure Compact Nucleic Acid Automated System (Roche Diagnostic, Germany) and plasma viral load of HIV-1 infected individuals was estimated using Cobas TaqMan Real Time PCR (Roche Molecular Systems, Piscataway, NJ) with a limit of detection of 34 RNA copies/ml. Due to the non-availability of a standard, reproducible HIV-2 viral load estimation assay and thus the inability to compare viral loads from HIV-1 infected individuals, we were unable to estimate the viral load of HIV-2 infected individuals.

**Table 1:** Clinical characteristics of participants

	HIV-1		HIV-2		Seronegative
	ART-naïve	ART- receiving^d^	ART-naïve	ART- receiving^d^	
Number of participants	24	19	16	19	33
Age^a^ (Years)	39 (18–57)	41.50 (30–50)	46 (23–55)	45.5 (20–59)	30 (21–55)
CD4 Cell Count^a^ (Cells/ul)	554 (210–990)	625 (372–1508)	813^b^ (504-1641)	441^c^ (158-1107)	NA
Viremia^a^ (RNA Copies/ml)	14,199 (203–400,644)	< 34	NA	NA	NA
Duration between diagnosis and sampling^a^ (Years)	1.4 (0.8–3.5)	4 (2–6)	1 (0.5–3)	3 (1–7)	–

^**a**^Data are expressed as the median (range)

^b^Absolute CD4 count for ART-naïve HIV-2 infected individuals was significantly higher than that for HIV-1 infected participants (unpaired t-test with Welch’s correction; *p* < 0.01)

^c^Absolute CD4 count for ART-naïve HIV-2 infected individuals was significantly higher than that for ART-treated HIV-2 infected participants (unpaired t-test with Welch’s correction; p < 0.01)

*NA* not applicable

^d^ART regimen for HIV-1 infected individuals was Zidovudine (AZT) + Lamivudine (3TC) + Nevirapine (NVP) and regimen for HIV-2 infected individuals was Zidovudine (AZT) + Lamivudine (3TC) + Lopinavir /Ritonavir (LPV)

All individuals that were included in ART receiving groups of either infection were on ART for at least 1 year. For HIV-1 infected individuals, range with median duration was (1–3) 1.8 years and for HIV-2 infected individuals, range with median duration was (1–3) 2 years

### Immunophenotypic analysis of T cell subsets

For immunophenotypic staining peripheral blood collected in EDTA vacutainer were stained with appropriate fluorochrome-conjugated surface antibodies, including anti-CD3 (Clone:SK7), anti-CD4 (Clone:RPA-T4), anti-CD8 (Clone:SK1), anti-CD25 (Clone:M-A251), anti-CD127 (Clone: HIL-7R-M21), anti-HLADR (Clone:L243), anti-CD38 (Clone:HIT2), anti-CD45RA (Clone:HI100) and anti-granzyme (Clone:GB11); purchased from either BD Biosciences or Biolegend. Intracellular staining for Granzyme was performed according to manufacturer’s instructions (BD Cytofix/Cytoperm™ Plus, Catalog No.-554,715) after surface staining with specific surface marker antibodies. The samples were processed on the same day of sampling for ex-vivo staining and ICCS Assay for Granzyme detection. Flow cytometric acquisition and analysis were performed on at least 50,000 acquired events (gated on lymphocytes) on a BD ACCURI C6 flow cytometer (BD Biosciences). The 670LP and 675/25 filters were used to measure the fluorescence corresponding to anti-CD25 and anti-CD127 antibodies respectively. The stochastic % standard deviation (SD) of MFI for 670LP and 675/25 filter was calculated using Spherotech 6-Peak (Cat No-653145, BD Biosciences) and 8-peak (Cat No-653144, BD Biosciences) Validation Beads and was found to be 3.78 and 2.43% respectively for the period during which study samples were acquired. Data Analysis was performed using FlowJo (Tree Star Inc., Ashland, Oregon, USA).

### Statistical analysis

Statistical analysis was performed using GraphPad Prism version 5.00 (GraphPad Software, San Diego, California, USA). The data are presented as scatter plots, with bars indicating median values and groups were compared using unpaired t-test with Welch’s correction 95% confidence interval. The prospective data was analysed using Repeated measures ANOVA and non-parametric paired T test (Wilcoxon matched). Non parametric Spearman’s correlation coefficient was used to assess the correlation between two variables. *P* values less than 0.05 were considered significant.

## Results

### Distribution of CD4^+^T cell subsets defined on the basis of expression of CD127 (IL-7R) and CD25 (IL-2Rα) in both HIV-1 and HIV-2 infected ART-naïve individuals

**Fig. 1:**
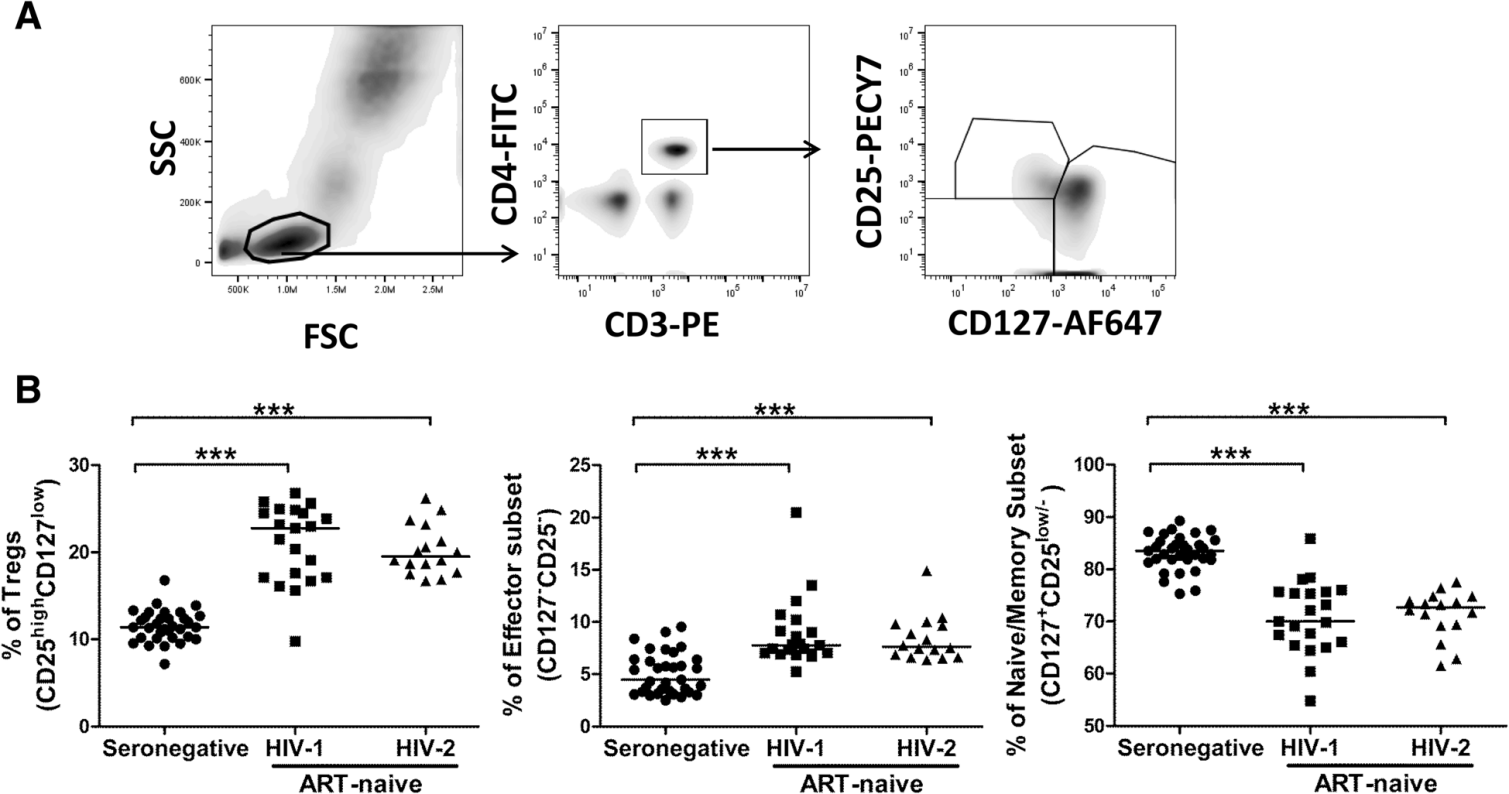
Identification of dysregulation in CD4^+^T cell subsets based on the expression of CD127 (IL-7R) and CD25 (IL-2Rα). **a** Gating strategy for defining subsets of CD4^+^ T cells using CD127 and CD25. Cells were gated based on characteristic light scatter properties FSC against SSC, followed by gating on CD4^+^ T cells. Thereafter based on expression of CD127 and CD25, CD4^+^T cells were further demarcated as naive/memory (CD127^+^CD25^low/−^), effector (CD127^−^CD25^−^) and Tregs (CD25^high^CD127^low^). **b** Comparison of frequency of CD4^+^ T cells subsets in ART-naïve HIV-1 (*n* = 21), HIV-2 (*n* = 16) infected individuals and seronegative individuals (*n* = 33). Statistical significance was evaluated by unpaired t test; *, *p* < 0.05; **, *p* < 0.01; and ***, *p* < 0.001

When the relative proportions of these CD4^+^T cell subsets were examined in ART-naïve HIV-1 and HIV-2 infected individuals, we observed a significant increase in the frequency of the Tregs (CD25^high^CD127^low^) subset (P = < 0.0001 for HIV-1; P = < 0.0001 for HIV-2) and effector memory (CD127^−^CD25^−^) subset (P = < 0.0001 for HIV-1; P = < 0.0001 for HIV-2), and a decline in the fraction of naive/central memory (CD127^+^CD25^low/−^) T cell subset (P = < 0.0001 for HIV-1; P = < 0.0001 for HIV-2) in both HIV-1 and HIV-2 infected individuals as compared to seronegative controls. Also, the frequency of these CD4^+^T cell subsets was found to be similar in both ART-naïve HIV-1 and HIV-2 infected individuals (Fig. 1).

To assess the association of these CD4^+^T cell subsets with disease progression under ART-naïve conditions in both HIV-1 and HIV-2 infected individuals, we correlated the frequency of these subsets with absolute CD4 count. Tregs (CD25^high^CD127^low^) subsets and effector memory (CD127^−^CD25-) cells were found to be negatively correlated and naive/memory (CD127^+^CD25^low/−^) T cell subset was found to be positively correlated with absolute CD4 count in both HIV-1 and HIV-2 study groups (Table 2). In addition, the degree of expression per-cell was estimated using median fluorescence intensity (MFI) for both CD25 and CD127 on CD4^+^T cell subsets and compared across groups. No significant difference was observed in the MFI of either of these markers in CD4^+^T cell subsets between HIV infected individuals (MFI of CD25: *P* = 0.1537 for HIV-1; *P* = 0.1380 for HIV-2, MFI of CD127: *P* = 0.1345 for HIV-1; *P* = 0.1012 for HIV-2) and seronegative controls (Fig. 4b). Furthermore, when the MFI for both markers was correlated with absolute CD4 count, no significant correlation with disease progression was observed (Table 2). To address any effect of unequal sample size between seronegative group and HIV infected groups on statistical analysis, a bootstrapping analysis was carried out (Additional file 2: Figure S2) which showed the same observations as reported above. Also to address any effect of unmatched ages of participant groups on statistical analysis, age matching was performed (Additional file 3: Figure S3).This had no impact on reported observations.

**Table 2:** Association of CD4 T cell subsets, MFI of CD25 and CD127, Level of immune activation and level of cytotoxicity with absolute CD4 count across the groups

	HIV-1				HIV-2			
	ART-naïve		ART-receiving		ART-naïve		ART-receiving	
	r	*P* value	r	*P* value	r	*P* value	r	*P* value
% Treg cells	−0.6849	**0.0034**	− 0.2905	0.3357	− 0.8505	**< 0.0001**	−0.6299	**0.0067**
% Effector CD4+ T cells	−0.4059	0.1188	−0.5337	0.0603	−0.5541	**0.0321**	−0.4608	0.0627
% Naïve/ memory CD4+ T cells	0.6435	**0.0071**	0.4512	0.1218	0.7379	**0.0017**	0.5564	**0.0204**
MFI Of CD25 within Tregs	−0.05527	0.8389	0.4182	0.1550	−0.3825	0.1594	0.1864	0.4738
MFI of CD127 within naive/memory CD4+ T cells	−0.2424	0.3656	0.3901	0.1876	0.1126	0.6895	0.4856	**0.0482**
% CD4+ HLADR+ CD38+ T cells	−0.7333	**0.0311**	NA		−0.2857	0.3440	NA	
% CD8+ HLADR+ CD38+ T cells	−0.7500	**0.0255**	NA		−0.3571	0.2309	NA	
% CD4+ GrzB+ T cells	0.8693	**0.0022**	NA		0.04945	0.8725	NA	
% CD8+ GrzB + T cells	−0.7591	**0.0149**	NA		−0.3736	0.2086	NA	

**Bold** font if significant *p* value; *NA* Not available

### Immune activation, under ART-naïve conditions, in both HIV-1 and HIV-2 infection

**Fig. 2:**
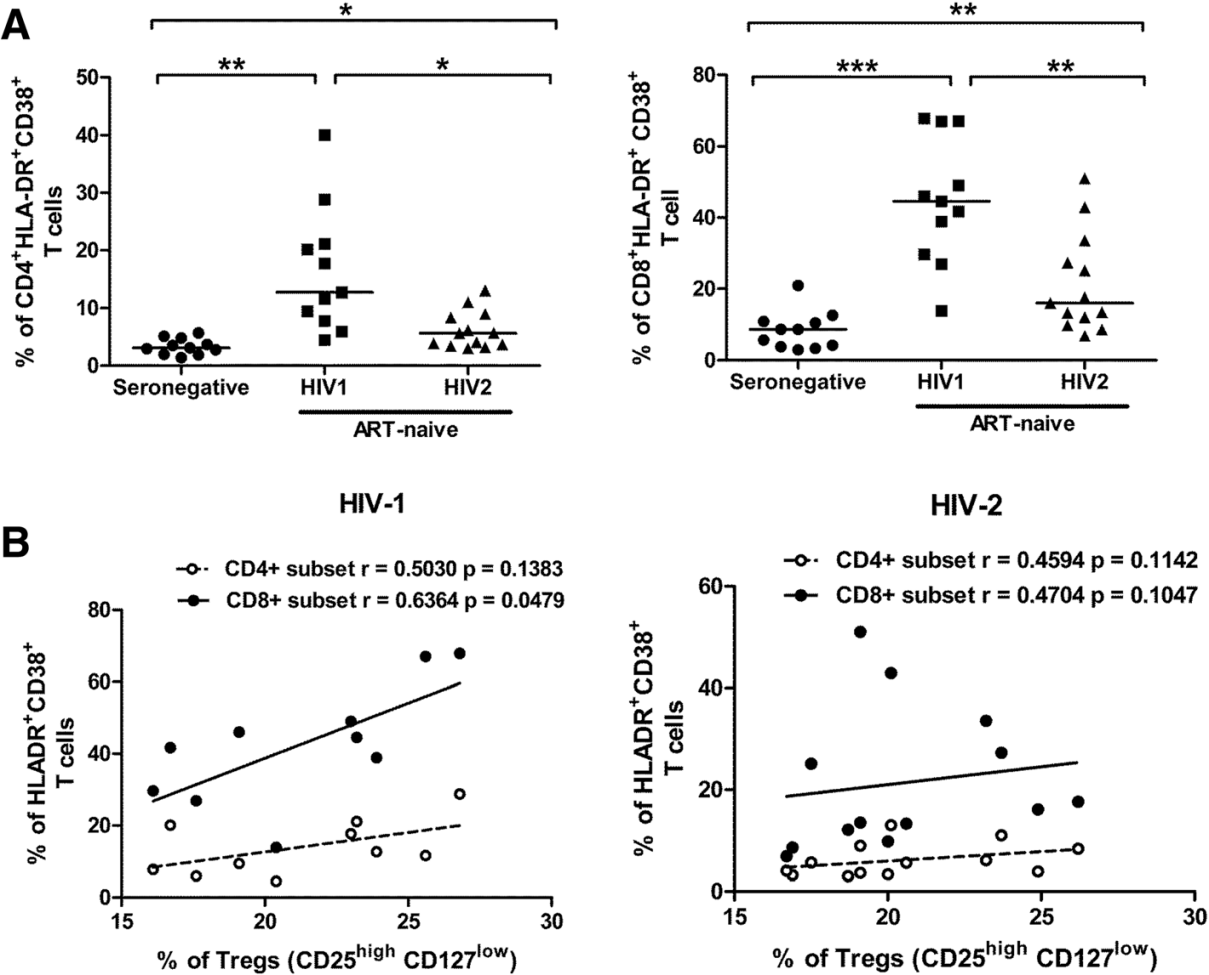
Level of Immune activation in HIV-1 and HIV-2 chronic infection. **a** Comparison of expression of immune activation markers (CD38&HLA-DR) on CD4^+^T and CD8^+^T cells among ART-naïve HIV-1 (*n* = 11), HIV-2 (*n* = 13) infected individuals and seronegative individuals (n = 11). Statistical significance was evaluated by unpaired t test; *, *p* < 0.05; **, *p* < 0.01; and ***, *p* < 0.001. The frequencies of activated cells are those observed within CD4^+^T and CD8^+^T cell gates as outlined in Additional file 4 Figure S4. **b** Correlation analysis between frequency of CD4^+^ and CD8^+^ activated T cells and Tregs cells in both ART-naïve HIV-1(*n* = 11) and HIV-2 (*n* = 13) infected individuals. Spearman’s correlation test was used to determine the correlation coefficient (r) and the significance (*p* < 0.05).

In our study, the frequency of activated (CD38^+^HLA-DR^+^) cells in both CD4^+^T (*P* = 0.0027 for HIV-1; *P* = 0.0119 for HIV-2) and CD8^+^T cell (P = < 0.0001 for HIV-1; *P* = 0.0071 for HIV-2) compartments was found to be elevated in both HIV-1 and HIV-2 infected groups as compared to seronegative controls. We also observed elevated levels of activation (CD38^+^HLA-DR^+^ cells) in HIV-1 infected individuals as compared to HIV-2 infected individuals in both CD4^+^T (*P* = 0.0117) and CD8^+^T cell (*P* = 0.0022) compartments (Fig. 2a). The elevated level of activation (CD38^+^HLA-DR^+^ cells) in the CD4^+^T and CD8^+^T cell compartment negatively correlated with absolute CD4 count in HIV-1 infected individuals, but not with that in HIV-2 infected participants (Table 2).

Recent studies have suggested that Tregs, might play a role in the dynamics of immune activation during HIV-1 infection [23–25]. However no study has assessed relationship between Tregs and the activation (CD38^+^HLA-DR^+^ cells) status of CD4^+^ or CD8^+^T cells in HIV-2 infection. On performing this analysis, we observed a positive correlation (though not significant) between the proportions of activated CD4^+^T cells and Tregs in both HIV-1 (r = 0.5030, *P* = 0.1383) and HIV-2 infected (r = 0.4594, *P* = 1142) study groups. Interestingly, a clear and significant positive correlation was observed between activated CD8^+^T cells and Tregs among HIV-1 infected individuals (r = 0.6364, *P* = 0.0479) and not in those infected with HIV-2 (r = 0.4704, *P* = 0.1047) (Fig. 2b).

### Granzyme-B expression in CD4^+^ and CD8^+^T cells in ART-naïve infected individuals

**Fig. 3:**
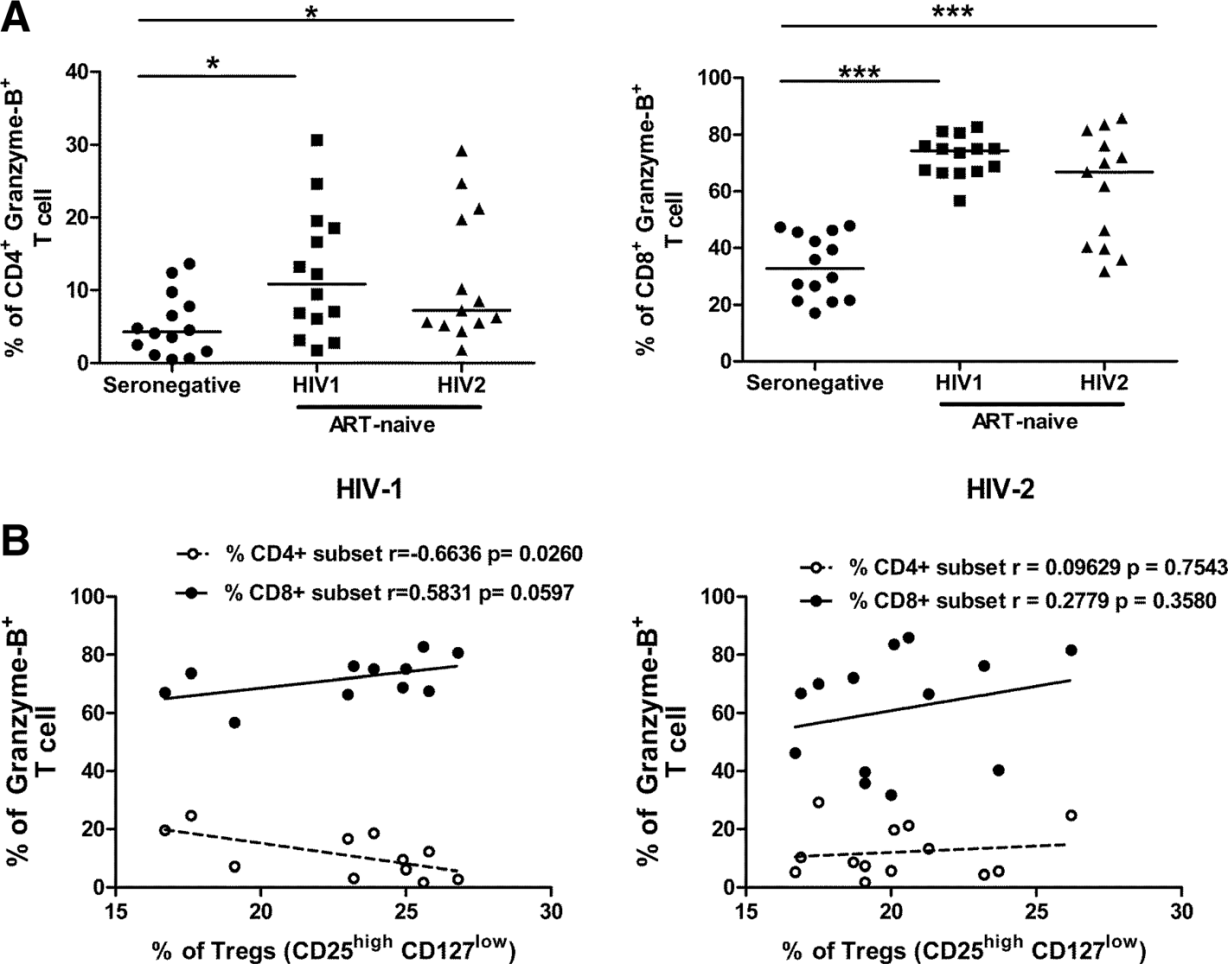
Expression of Granzyme B in CD4^+^T and CD8^+^T cells. **a** Comparison level of GrzB from CD4^+^ T and CD8^+^T cells among ART-naïve HIV-1(*n* = 14), HIV-2 (*n* = 13) infected individuals and seronegative individuals (*n* = 14). Statistical significance was evaluated by unpaired t test; *, *p* < 0.05; **, *p* < 0.01; and ***, *p* < 0.001. Gating strategy outlined in Additional file 4: Figure S4. **b** Correlation analysis between level of GrzB and frequency of Tregs cells in both ART-naïve HIV-1 (*n* = 10) and HIV-2 (*n* = 13) infected individuals.Spearman’s correlation test was used to determine the correlation coefficient (r) and the significance (*p* < 0.05)

We observed that, the level of GrzB expression in CD4^+^T (*P* = 0.0138 for HIV-1; *P* = 0.0355 for HIV-2) and CD8^+^T cells (P = < 0.0001 for HIV-1; *P* = 0.0003 for HIV-2) was substantially higher in both HIV-1 and HIV-2 infected individuals compared to seronegative controls. However, the level of GrzB expression in CD4^+^T (*P* = 0.8150) and CD8^+^T cells (*P* = 0.0673) was found to be similar in both HIV-1 and HIV-2 infected individuals (Fig. 3a).

To investigate whether levels of total circulating cytotoxic T cells were associated with disease progression, the correlation between levels of GrzB expressing cells within the CD4^+^T and CD8^+^T cell subsets with absolute CD4 count was carried out. As shown in Table 2, a significant positive correlation was observed between levels of GrzB expressing CD4^+^T cells and absolute CD4 count in HIV-1 infected individuals. Additionally, a significant negative correlation between levels of GrzB expressing CD8^+^T cell and absolute CD4 count in HIV-1infected individuals was observed. However this correlation was not observed in HIV-2 infected individuals. Furthermore, when we assessed the association of Tregs and cytoxicity, as expected, a significant negative correlation (r = − 0.6636, *P* = 0.0260) was detected between frequency of GrzB producing CD4^+^T cells and frequency of Tregs in HIV-1 infected study group (Fig. 3b). Also, positive correlation was detected between frequency of GrzB producing CD8^+^T cells and frequency of Tregs in the HIV-1 infected study group (r = 0.5831, *P* = 0.0597). However, no significant association was detected between the level of cytotoxicity and Tregs in HIV-2 infected individuals (r = 0.0962, *P* = 0.7543 for GrzB^+^CD4^+^T cells; r = 0.2779 *P* = 0.3580 GrzB^+^CD8^+^T cells).

### Effect of antiretroviral therapy on CD4^+^T cell subset defined on the basis of expression of CD25 and CD127

We sought to determine the frequency of CD4^+^T cell subsets in the whole blood of individuals who were on extended (> 1 year), successful (HIV-1 viral load < 34copies/ml) uninterrupted ART in comparison to ART-naïve individuals and seronegative individuals.

**Fig. 4:**
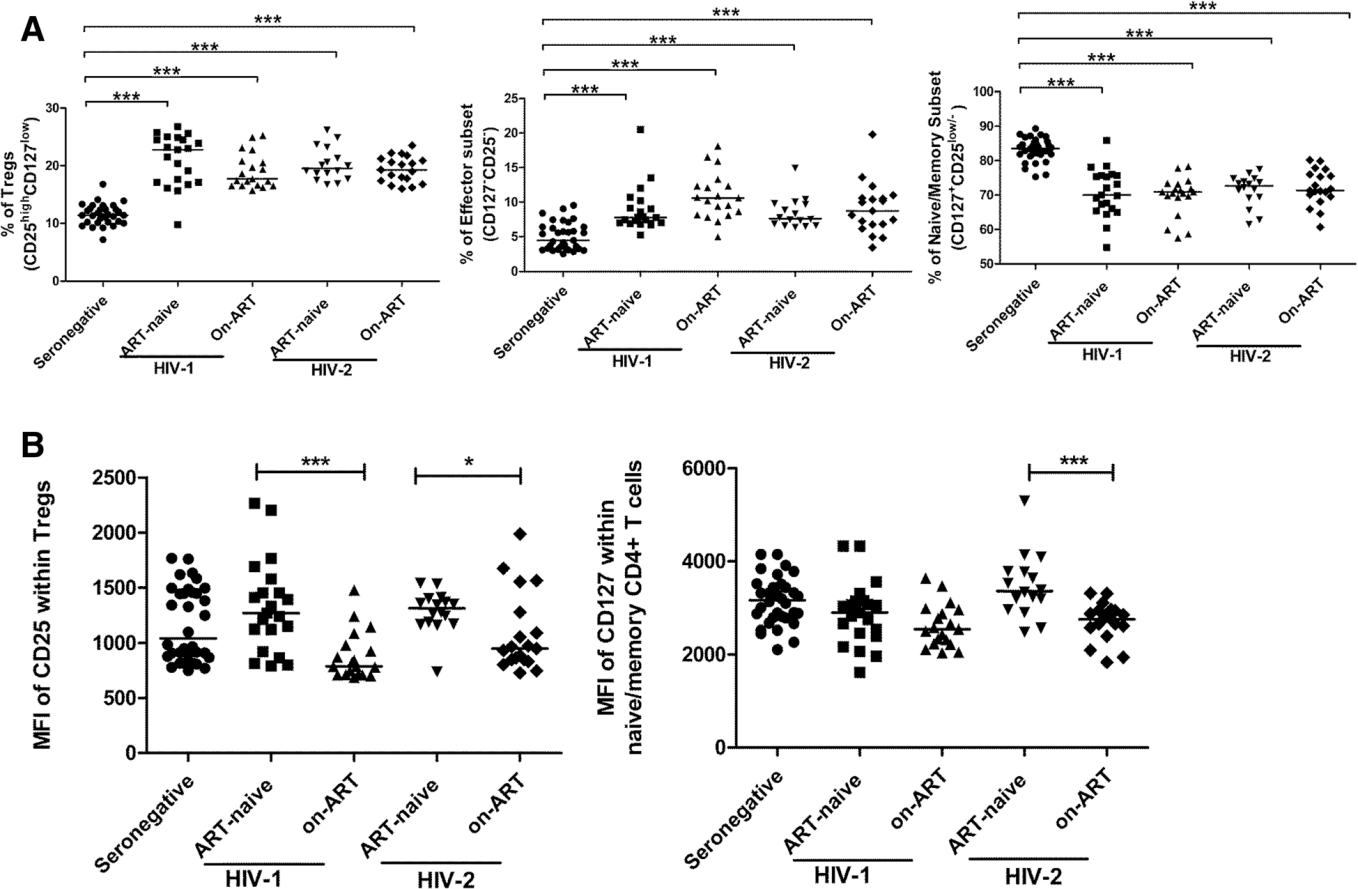
Effect of Antiretroviral therapy on CD4^+^T cell subset defined on the basis of expression of CD25 and CD127. **a** Comparison of CD4^+^ T cells subsets in ART-naïve HIV-1 (*n* = 21), ART-treated HIV-1 (*n* = 19), ART-naïve HIV-2 (n = 16), ART-treated HIV-2 (*n* = 19) infected individuals and seronegative individuals (*n* = 33). **b** Comparison of MFI of CD25 and CD127 in ART-naïve HIV-1 (*n* = 21), ART-treated HIV-1 (*n* = 19), ART-naïve HIV-2 (*n* = 16), ART-treated HIV-2 (*n* = 19) infected individuals and seronegative individuals (*n* = 33). Statistical significance was evaluated by unpaired t test; *, *p* < 0.05; **, *p* < 0.01; and ***, *p* < 0.001

We observed no alteration in the frequency of the Tregs (CD25^high^CD127^low^) subset, effector memory (CD127^−^CD25^−^) subset and naive/memory (CD127^+^CD25^low/−^) T cell subset between ART- receiving and ART-naïve HIV-1 and HIV-2 infected individuals. However, we observed significantly increased levels, similar to those observed in ART-naïve infected individuals, in the frequency of the Tregs (CD25^high^CD127^low^) subset (P = < 0.0001 for HIV-1; P = < 0.0001 for HIV-2), effector memory (CD127^−^CD25^−^) subset (P = < 0.0001 for HIV-1; *P* = 0.0002 for HIV-2), and decline in the fraction of naive/memory (CD127^+^CD25^low/−^) T cell subset (P = < 0.0001 for HIV-1; P = < 0.0001 for HIV-2) compared to seronegative controls (Fig. 4a). When the MFI of CD25 within Tregs was evaluated, we observed significant reduction in ART- receiving HIV infected individuals (P = 0.0002 for HIV-1; *P* = 0.0454 for HIV-2) compared to that in ART-naïve individuals. Moreover, the MFI of CD127 within naive/memory (CD127^+^CD25^low/−^) T cell subset was also observed to be significantly lower in ART- receiving HIV-2 infected individuals (*P* = 0.0007) compared to ART-naïve HIV-2 infected individuals (Fig. 4b). A similar trend (*p* = 0.06) was observed for HIV-1 infected individuals. The % reduction in MFI of CD25 within Tregs was 38 and 27.6% for HIV-1 and HIV-2 infected individuals respectively when compared to the MFI of corresponding ART-naïve groups. Also the % reduction in MFI of CD127 within naive/memory (CD127^+^CD25^low/−^) T cell subset was 17.9% for ART- receiving HIV-2 infected individuals compared to ART-naïve group. These changes in MFI were clearly higher than stochastic variation of fluorescence intensity calculated for the instrument during the period of study (see methods).

When the association between absolute CD4 count and frequency of CD4^+^T subsets in ART- receiving HIV-1 and HIV-2 infected individuals was examined, a significant negative correlation was observed between the absolute CD4 count and the frequency of the Tregs (CD25^high^CD127^low^) and a significant positive correlation was observed between the absolute CD4 count and the frequency of the naive/memory (CD127^+^CD25^low/−^) T cell subset in ART- receiving HIV-2 infected individuals (Table 2). No correlation was observed between absolute CD4 count and frequency of CD4^+^T cell subsets in ART- receiving HIV-1 infected individuals. Also the MFI of CD127 within naive/memory (CD127^+^CD25^low/−)^ T cell subset and MFI of CD25 within Tregs in ART- receiving HIV-1 infected individuals showed no correlation with absolute CD4 count. However MFI of CD127 within naive/memory (CD127^+^CD25^low/−^) T cell subset showed significant positive correlation with absolute CD4 count in ART- receiving HIV-2 infected individuals. No correlation was observed between absolute CD4 count and MFI of CD25 within Tregs in ART- receiving HIV-2 infected individuals (Table 2).

**Fig. 5:**
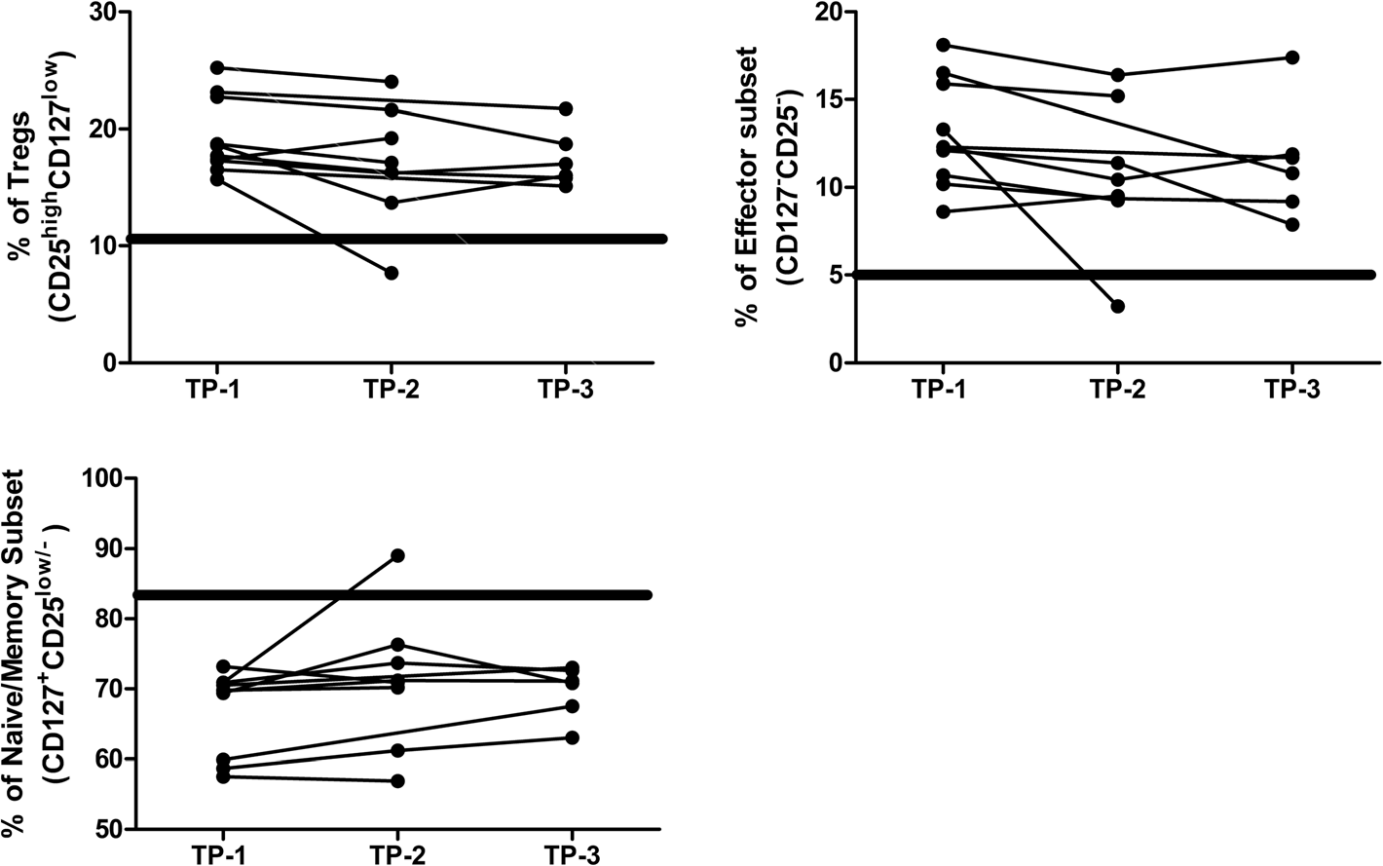
Prospective data analysis of CD4^+^T cell subsets. Graphical presentation of CD4^+^T cell subset frequencies from 10 ART-receiving (for more than one year) HIV-1 infected individuals at 3 months (TP-2) and 18 months (TP-3) after enrolment (TP-1). Bold rectangular line represents the mean ± SEM frequency of corresponding CD4^+^ T cell subset in seronegative individuals (n = 33)

Further, we followed 10 ART-receiving (for more than one year) HIV-1 infected individuals at 3 months (TP-2) and 18 months (TP-3) after enrolment (TP-1) in this study to evaluate whether extended ART was able to restore the dysregulated CD4^+^Tcell subset frequencies to normal levels. Due to difficulties in follow up, we were unable to follow all individuals at each time point. As presented in Table 3A, CD4^+^T cell subset data was available for TP-1 and TP-2 from 8 individuals (Group-1), for TP-1 and TP-3 from 2 individuals (Group-2) and for TP-1, TP-2 and TP-3 from 4 individuals (Group-3). Assessment of the relative proportions of these three CD4^+^T cell subsets was carried out for Group-1 and Group-3, where as shown in Table 3B and C respectively, no significant change was observed. Figure 5 represents the graphical presentation of the data.

**Table 3:** Prospective data analysis of CD4 T cell subsets

**A** Sampling and analysis of all prospective data (*n* = 10)									
ART-receiving HIV-1 infected individuals no.	**% of Treg (CD25** ^**high**^ **CD127** ^**low**^ **)**			**% of Effector subset** **(CD127** ^**−**^ **CD25** ^**−**^ **)**			**% of Naïve/Memory subset(CD127** ^**+**^ **CD25** ^**low/−**^ **)**		
	TP-1	TP-2	TP-3	TP-1	TP-2	TP-3	TP-1	TP-2	TP-3
1	17.4	19.20	SNA	8.61	9.51	SNA	73.20	70.90	SNA
2	22.7	21.60	18.7	18.10	16.40	17.40	58.70	61.20	63.00
3	16.5	SNA	15.1	12.30	SNA	11.70	70.50	SNA	73.00
4	25.2	24.00	SNA	15.90	15.20	SNA	57.50	56.90	SNA
5	18.6	13.70	16.0	10.20	9.36	9.19	70.90	73.70	72.60
6	17.7	16.30	15.8	12.10	11.40	7.88	69.70	71.20	71.10
7	17.3	16.20	17.0	12.30	10.45	11.90	69.40	76.30	70.80
8	18.7	17.10	SNA	10.70	9.26	SNA	69.80	70.20	SNA
9	23.1	SNA	21.7	16.50	SNA	10.80	59.90	SNA	67.50
10	15.7	7.65	SNA	13.30	3.21	SNA	70.90	89.00	SNA
**B** Comparison of CD4^+^ T cells subsets in ART-treated HIV-1 infected individuals (*n* = 8) from group 1 (TP-1 and TP-2)									
Non-parametric paired T test (Wilcoxon matched paired test)	**% of Treg (CD25** ^**high**^ **CD127** ^**low**^ **)**			**% of Effector subset (CD127** ^**−**^ **CD25** ^**−**^ **)**			**% of Naïve/Memory subset (CD127** ^**+**^ **CD25** ^**low/−**^ **)**		
*P* value	0.1094			0.0547			0.1094		
*P* value summary (*P* < 0.05)	ns			ns			ns		
**C** Comparison of CD4^+^ T cells subsets in ART-treated HIV-1 infected individuals (*n* = 4) from group 3 (TP1, TP2 and TP3)									
Repeated Measures ANOVA	**% of Treg (CD25** ^**high**^ **CD127** ^**low**^ **)**			**% of Effector subset (CD127** ^**−**^ **CD25** ^**−**^ **)**			**% of Naïve/Memory subset** **(CD127** ^**+**^ **CD25** ^**low/−**^ **)**		
*P* value	0.0978			0.2192			0.0724		
*P* value summary (*P* < 0.05)	ns			ns			ns		

*SNA* Sample not available, *TP1–3* Time points at enrolment, 3 and 18 months follow up respectively; Nos 1, 2, 4, 5, 6, 7, 8 and 10, Group-1 (TP-1 and TP-2); Nos. 3 and 9, Group-2 (TP-1 and TP-3); Nos. 2, 5, 6 and 7, Group-3 (TP-1, TP-2 and TP-3)

## Discussion

This is the first study evaluating concurrently the dynamics of the T cell compartment using homeostatic markers such as CD25 (IL-2Rα) and CD127 (IL-7R), level of immune activation and circulating cytotoxic T cell levels in HIV-2 infected individuals. Furthermore, the dynamics, in terms of these markers of the CD4^+^T cell compartment has been addressed separately in the absence of viremia due to effective ART in individuals infected with HIV-1 or HIV-2 in the Indian population for the first time.

Progressive decline of CD4^+^T cells following HIV infection is a hallmark of disease progression resulting in HIV associated immune dysfunction [26]. In this study, both ART-naïve HIV-1 and HIV-2 infected individuals showed apparent dysregulation (altered CD25 and CD127 expression) in CD4^+^T cell subsets and this dysregulation was found to be associated with disease progression. These results suggest an altered cytokine network with respect to IL-2 and IL-7 homeostasis present in HIV infected individuals. Also, the increased frequency of Tregs (CD25^high^CD127^low^) and effector memory (CD127^−^CD25^−^) subsets seen in both infections within this study may reflect an increased level of immune activation present in these individuals as consequence of viremia in the absence of ART. The significantly lower frequency of the naive/memory (CD127^+^CD25^low/−^) T cell subset is congruent with loss of central memory CCR5 expressing CD4^+^T cells as they represent the primary target of HIV infection. Our results with respect to increased frequency of Tregs in ART-naïve HIV infected individuals compared to seronegative individuals are in agreement with some previous reports describing HIV-1 clade B and HIV-2 clade A infection [24, 27–29]. However, our result with respect to increased frequency of Tregs in ART-naïve HIV-1 infected individuals are not in agreement with some reports [11, 27] that used PBMC staining and were unable to detect differences in frequency of Tregs defined as CD4^+^CD25^bright^ [27] and CD25^high^CD127^low^ [11] compared to seronegative individuals.

Effective Antiretroviral therapy (ART) blunts viral replication and should result in an increase of the CD4^+^T cell count in peripheral blood, a phenomenon known as immune reconstitution [30]. However the data available so far is limited and conflicting with respect to observed differences in the frequency of Tregs in HIV-1 infection followed by extensive ART [30–34]. No study has assessed the effect of ART on CD4^+^T subset defined on the basis of expression of CD25 and CD127 in HIV-1 and HIV-2 infection. Analysing the effect of ART on these CD4^+^T cell subsets revealed that ART-receiving HIV infected individuals showed similar frequencies of circulating CD4^+^T cell subsets to those in ART-naïve HIV infected individuals and were higher than seronegative individuals. Also longitudinal assessment of these CD4^+^T cell subsets in ART- receiving HIV-1 infected individuals revealed that they were maintained at high levels over an extended period of ART in the absence of detectable viral replication. These results suggest that ART was not able to rectify a disruption which was occurring in immune homeostasis with respect to IL-2Rα (CD25) and IL-7R (CD127) expressing CD4^+^T cells in both HIV-1 and HIV-2 infection. Though, we recognise that this data would be stronger if it included baseline frequency value of these subsets, which was not available, before the initiation of ART.

Median fluorescent intensity is ‘per-cell’ basis expression of a marker on a cell and alteration in MFI’s could also indicate the functional state for that marker. This paradigm has been supported by studies reporting CD25 expression and functionality of Treg cells [35]. Thus, we evaluated the MFI of both CD127 as well as CD25 in CD4^+^T cell subsets to ascertain if these correlated with disease progression as was found to be the case for frequencies of the subsets that are defined by them. The MFI of CD127 within naive/memory (CD127^+^CD25^low/−^)T cell subset and MFI of CD25 within Tregs (CD25^high^CD127^low^) in ART-naïve HIV infected individuals showed no association with disease progression which suggests that frequency of CD4^+^ T cell subsets defined by their expression are better predictors of disease progression compared to their per cell expression on these subsets.

Though there were no differences in the frequency of CD4^+^T cell subsets in ART- receiving HIV infected individuals compared to ART-naïve HIV infected individuals, we observed reduced per cell expression of CD25 and CD127 in ART- receiving HIV infected individuals compared to ART-naïve HIV infected individuals. This suggests that ART was able to alter the expression of IL-2Rα and IL-7R following successful virological control. While this is indicative of possible or impending immune restoration, it is noteworthy that it was the frequency of CD4^+^ T cell subsets and not MFI of CD25 or CD127 within these subsets that continued to show stronger correlation with disease progression (absolute CD4 count) in HIV infected individuals undergoing virologically effective ART.

Overall, we showed that dysregulation of CD4^+^T cell subsets occurred in infected individuals irrespective of the presence or absence of ART suggesting that the relative disruption in cytokine networks with respect to IL-2 and IL-7 homeostasis caused by HIV infection is persistent in infected individuals and may not be rectifiable through ART alone. From a clinical management point of view in India, our work provides the rationale for exploring new therapeutic strategies for both HIV-1 and HIV-2 infection aimed at directly correcting the HIV-induced immune dysfunction. Also both IL-2 and IL-7 are being investigated as therapies to improve the immune system function in HIV-infected individuals [36–39].

Systemic immune activation is a feature of chronic HIV infection and determines the rate of disease progression [17, 18, 40]. HIV-2 infection has been reported to be associated with slower disease progression as compared to HIV-1.However the data available with respect to immune activation associated with infection is limited, conflicting and has reported on either CD4 or CD8 compartment in isolation [41–44]. The present study explored the level of immune activation in ART-naïve HIV-1 and HIV-2 infection in CD4^+^T and CD8^+^T cells.

Our findings demonstrate that in ART-naïve HIV-2 infected individuals, the level of immune activation in both CD4^+^T and CD8^+^ T cell compartment was lower compared to ART-naïve HIV-1 infected individuals and higher than seronegative individuals. While lower levels of immune activation could possibly be explained, in part due to lower viral load as has been reported in previously [41, 45], it is important to note that in our study, the levels of circulating Tregs were observed to be similar to those in ART-naïve HIV-1 infected individuals. Furthermore, these levels correlated inversely with absolute CD4 counts. Thus, these results suggest that the activation induced Treg response following both HIV-1 and HIV-2 infection while similar in magnitude may be more effective in ART-naïve HIV-2 infected individuals. This would explain the lower levels of immune activation as well as higher absolute CD4 counts observed and hence contribute to better disease progression. Interestingly, we also observed that the level of immune activation in CD4^+^T and CD8^+^T cell compartment was not associated with disease progression (absolute CD4 count) in ART-naïve HIV-2 infected study group in contrast to ART-naïve HIV-1 infected individuals. This data indicates that the observed lower levels of immune activation in ART-naïve HIV-2 infected individuals were not sufficiently high enough to drive persistent immune activation driven CD4^+^T cell depletion as observed in ART naïve HIV-1 infected individuals.

Release of Granzyme B, the principle Granzyme in CD8^+^cytotoxic T lymphocyte (CTL) and accompanying cytolytic destruction of target cells is believed to be one of the key immunological mechanisms for destruction of virally infected cells including those infected by HIV [46]. Also, with respect to HIV-1 and SIV infection, studies have delineated a role for CD4^+^ T cytotoxic responses in the prevention of acquisition, peak and set point viremia, control of rebounding virus in the absence SIV specific CD8^+^T cells and in effective CD8^+^T cell mediated responses against infected cells [47–50].

We report on the level of Granzyme-B expressing circulating CD4^+^T and CD8^+^T cells in ART-naïve HIV-2 infected individuals which, as in the case of ART-naïve HIV-1 infected individuals was observed to be higher compared to seronegative controls. These cytotoxic CD4^+^T and CD8^+^T cell may be assumed to include the HIV-specific cytotoxic T cells and indeed, correlation analysis between Granzyme-B expressing circulating CD4^+^T and CD8^+^T cells with absolute CD4 count showed a significant, though opposing correlation in HIV-1 infected individuals, suggesting that Granzyme-B expressing circulating CD4^+^T cells may be playing a protective role against disease progression in HIV-1 infection. Also, as expected, these CD4^+^T cell Granzyme-B responses correlated negatively with Treg frequencies in ART-naïve HIV-1 infected individuals. Intriguingly, no such correlation of Granzyme-B producing T cells with Treg frequency (or absolute CD4 count) and thus disease progression was observed in ART- naïve HIV-2 infected individuals. Taken together with the lack of correlation of activation levels and absolute CD4 counts in ART-naïve HIV-2 infected individuals, this data suggests a disparate pathogenesis for HIV-2 where possibly lower viral load and efficient Treg activity are associated with disease progression rather than activation and cytotoxic function of T cells. A future study, focusing on virus specific cytotoxic T cell responses would definitely provide a clearer picture.

## Conclusion

The study reports for the first time on levels of circulating Granzyme-B expressing CD4^+^T and CD8^+^T cells in chronic HIV-2 infection. Our data also highlights a possibly distinct pathogenesis mechanism operating in HIV-2 infected individuals who, despite having similar duration of infection have possibly lower viral load and efficient Treg activity. This would contribute to less activation driven CD4^+^T cell depletion and thus better disease progression. Furthermore, the observed disruption in cytokine networks with respect to IL-2 and IL-7 homeostasis caused by HIV infection is persistent in infected individuals and may not be rectifiable through ART alone. Our work also provides the rationale for exploring novel immunomodulatory therapeutic strategies for both HIV-1 and HIV-2 infection aimed at directly correcting the HIV-induced immune dysfunction.

## Additional files

Additional file 1: **Figure S1.** Validation CD4^+^CD25^high^CD127^low^ phenotype as Tregs. The lymphocyte population (A) was gated, followed by gating on CD4^+^ cells (B). Thereafter CD4^+^ lymphocytes, based on expression of CD25, CD127 and FOXP3 were demarcated as CD25^+^FOXP3^+^ (C) and CD25^high^CD127^low^ (D) population. As shown in E, when CD4^+^CD25^+^FOXP3^+^ (Tregs) population was overlaid on D, the Treg population (dark black dots) corresponds to the CD25^high^CD127^low^ population indicating that FOXP-3 is expressed by CD25^high^CD127^low^ population. Furthermore, the frequencies of CD4^+^CD25^+^FOXP3^+^ and CD4^+^CD25^high^CD127^low^ population obtained, through concurrent staining, from 9 seronegative individuals were compared and showed 97.6% correspondence between both these populations (F). (TIF 1132 kb)

Additional file 2: **Figure S2.** Bootstrapping exercise to address the effect of unequal sample size on statistical tests. A bootstrapping analysis was carried out to address the difference in numbers of recruited individuals. As the biggest disparity in numbers was between the seronegative (*N* = 33) and ART naïve HIV-2 group (*N* = 16), data from 16 individuals of the seronegative group, selected by randomisation (using MS Excel) 10 times, was used for comparison of subset frequencies independently. Following this analysis, where numbers of individuals in comparator groups were similar, we continued to observe a significant increase in the frequency of the Tregs (CD25highCD127low) and effector memory (CD127-CD25-) subset as well as a decline in the fraction of naive/central memory (CD127 + CD25low/−) T cell subset in HIV infected individuals as compared to seronegative controls (bootstrap 1 to 10). Statistical significance was evaluated by unpaired t test; *, *p* <  0.05; **, *p* <  0.01; and ***, *p* < 0.001. (TIF 7989 kb)

Additional file 3: **Figure S3.** Age match analysis to address effect of age on statistical analysis. Age and number of seronegative individuals were matched with HIV infected individuals by selecting the16 oldest individuals from seronegative group and their data was used for the comparison of subsets. The median age of the seronegative individuals was now 40 years and was very similar to the median age of HIV infected individuals as shown in A. Following this analysis, the observations with respect to CD4 + T cell subsets remained unchanged (Fig. B, C and D). With respect to data used for analysis of immune activation and cytotoxicity (Figs. 2 and 3) the age of individuals in each group was compared. The median age of all groups was in the range of 35-44 and 37-44 for immune activation (Fig. E) and cytotoxicity analysis (Fig. F) respectively with no significant statistical differences observed. Thus, both seronegative and HIV infected individuals, for these analyses, seem to be age matched appropriately. (TIF 6315 kb)

Additional file 4: **Figure S4.** Gating strategy for activation marker and granzyme-B. Gating strategy for activation marker: Cells were gated based on characteristic light scatter properties FSC against SSC, followed by gating on CD4^+^ T cells and CD8^+^T cells. Thereafter based on expression of HLADR and CD38, CD4^+^T (Fig. A) and CD8^+^T (Fig. B) cells were further demarcated as HLADR^+^CD38^+^ population. The HLADR^+^CD38^+^ population was reported as the activated population. The FMO control was used for gating positive population of CD38. Gating strategy for granzyme-B: The lymphocyte population was gated, followed by gating on CD4^+^ T cells and CD8^+^T cells. Thereafter based on expression of granzyme-B, CD4^+^T (Fig. C) and CD8^+^T (Fig. D) cells were further analysed for granzyme-B positivity (compared to FMO control) and this population was reported as cytotoxic T cells. At least 1, 00,000 events in the lymphocytes gate were acquired for granzyme-B detection. (TIF 2843 kb)

## Abbreviations

ART: Anti-retroviral therapy
CTL: Cytotoxic T cells
GrzB: Granzyme B
IL-2: Interleukin-2
IL-7: Interleukin-7
MFI: Median fluorescence intensity
PLHIV: People living with human immunodeficiency virus
