# Supplementary figures and images for: Immune signatures for HIV-1 and HIV-2 induced CD4+T cell dysregulation in an Indian cohort
Source: BMC Infect Dis. 2019 Feb 11;19:135. doi: 10.1186/s12879-019-3743-7 (PMC6371624; doi:10.1186/s12879-019-3743-7)

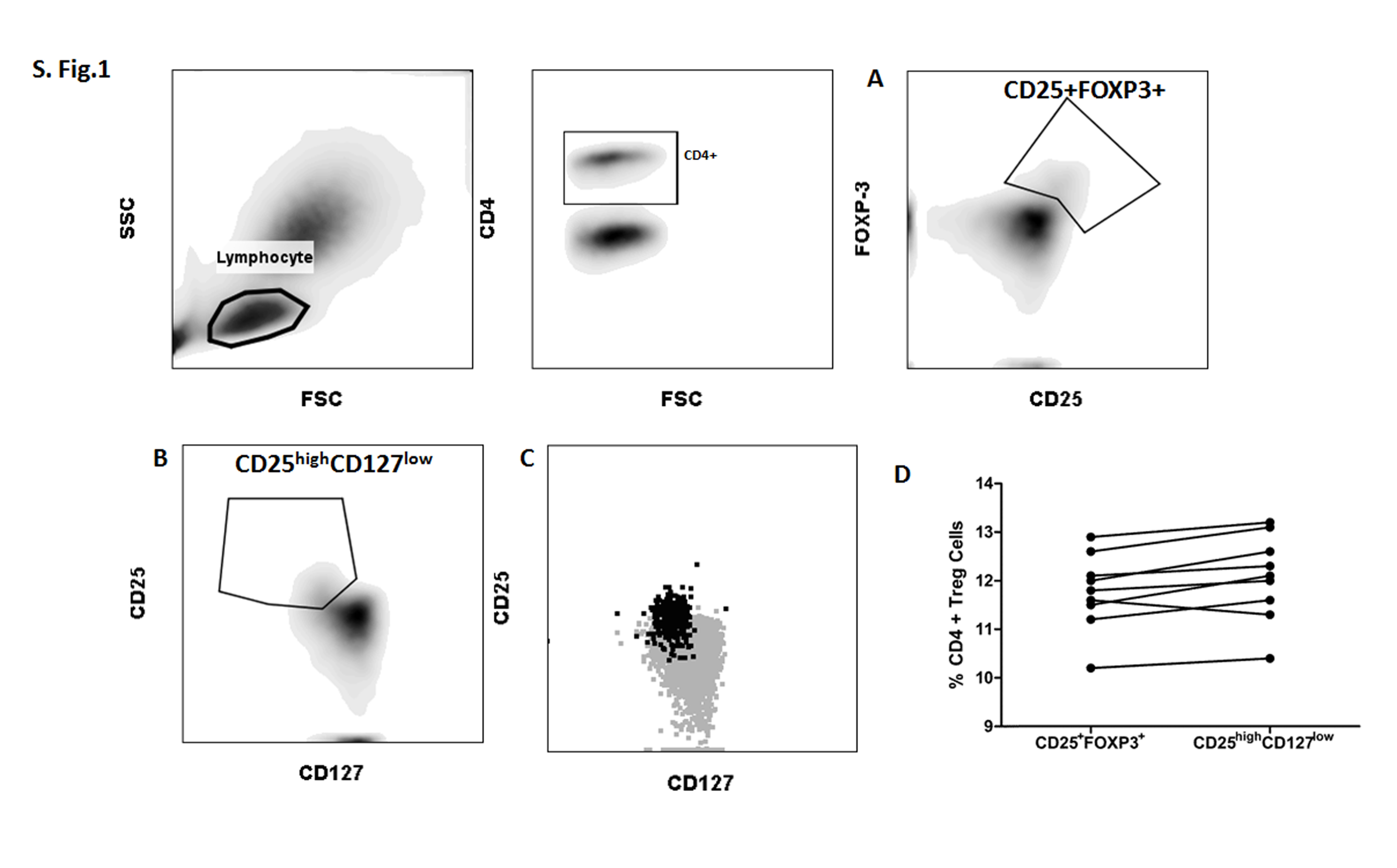

Supplement: Supplementary file 1 — Figure S1. Validation CD4+CD25highCD127low phenotype as Tregs. The lymphocyte population (A) was gated, followed by gating on CD4+ cells (B). Thereafter CD4+ lymphocytes, based on expression of CD25, CD127 and FOXP3 were demarcated as CD25+FOXP3+ (C) and CD25highCD127low (D) population. As shown in E, when CD4+CD25+FOXP3+ (Tregs) population was overlaid on D, the Treg population (dark black dots) corresponds to the CD25highCD127low population indicating that FOXP-3 is expressed by CD25highCD127low population. Furthermore, the frequencies of CD4+CD25+FOXP3+ and CD4+CD25highCD127low population obtained, through concurrent staining, from 9 seronegative individuals were compared and showed 97.6% correspondence between both these populations (F). (TIF 1132 kb) [file 12879_2019_3743_MOESM1_ESM.tif]

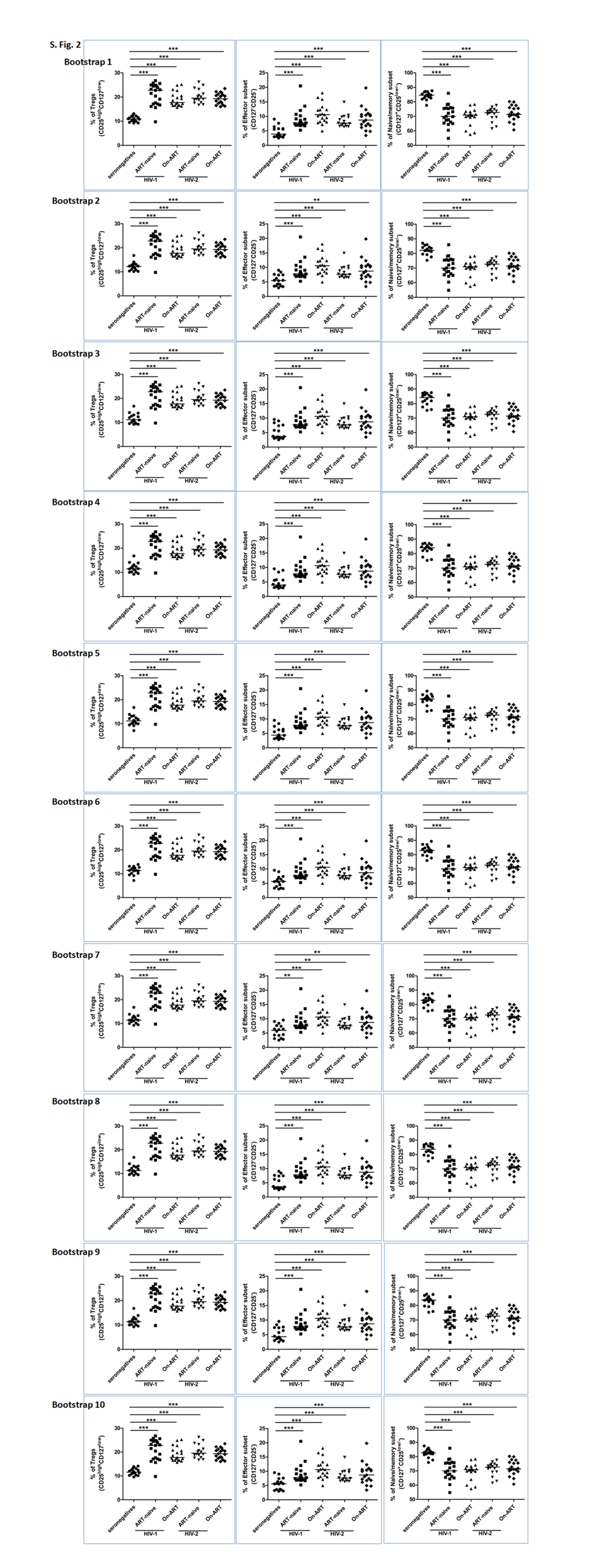

Supplement: Supplementary file 2 — Figure S2. Bootstrapping exercise to address the effect of unequal sample size on statistical tests. A bootstrapping analysis was carried out to address the difference in numbers of recruited individuals. As the biggest disparity in numbers was between the seronegative (N = 33) and ART naïve HIV-2 group (N = 16), data from 16 individuals of the seronegative group, selected by randomisation (using MS Excel) 10 times, was used for comparison of subset frequencies independently. Following this analysis, where numbers of individuals in comparator groups were similar, we continued to observe a significant increase in the frequency of the Tregs (CD25highCD127low) and effector memory (CD127-CD25-) subset as well as a decline in the fraction of naive/central memory (CD127 + CD25low/−) T cell subset in HIV infected individuals as compared to seronegative controls (bootstrap 1 to 10). Statistical significance was evaluated by unpaired t test; *, p < 0.05; **, p < 0.01; and ***, p < 0.001. (TIF 7989 kb) [file 12879_2019_3743_MOESM2_ESM.tif]

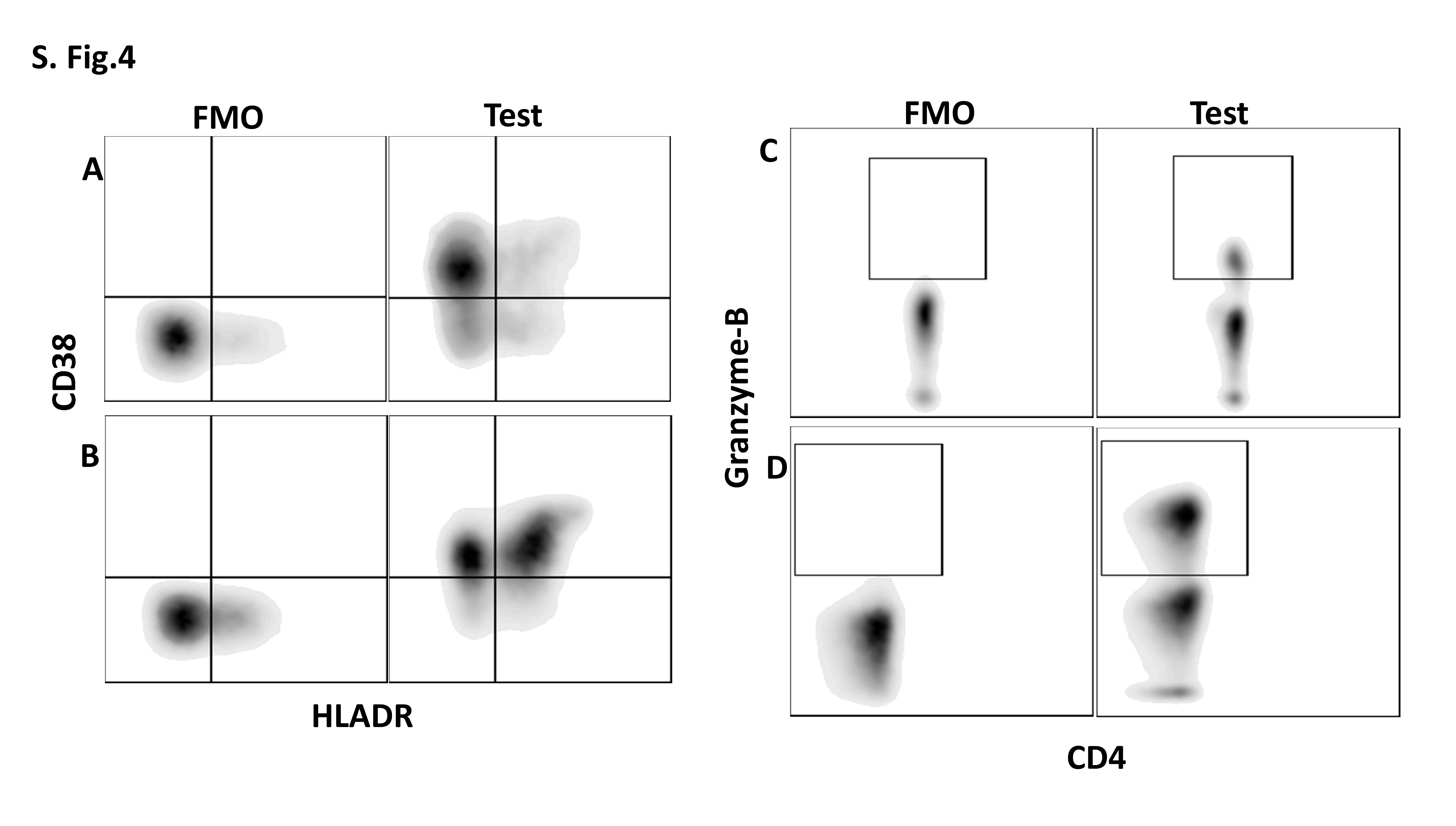

Supplement: Supplementary file 4 — Figure S4. Gating strategy for activation marker and granzyme-B. Gating strategy for activation marker: Cells were gated based on characteristic light scatter properties FSC against SSC, followed by gating on CD4+ T cells and CD8+T cells. Thereafter based on expression of HLADR and CD38, CD4+T (Fig. A) and CD8+T (Fig. B) cells were further demarcated as HLADR+CD38+ population. The HLADR+CD38+ population was reported as the activated population. The FMO control was used for gating positive population of CD38. Gating strategy for granzyme-B: The lymphocyte population was gated, followed by gating on CD4+ T cells and CD8+T cells. Thereafter based on expression of granzyme-B, CD4+T (Fig. C) and CD8+T (Fig. D) cells were further analysed for granzyme-B positivity (compared to FMO control) and this population was reported as cytotoxic T cells. At least 1, 00,000 events in the lymphocytes gate were acquired for granzyme-B detection. (TIF 2843 kb) [file 12879_2019_3743_MOESM4_ESM.tif]
